# Contemporary European practice in transcatheter aortic valve implantation: results from the 2022 European TAVI Pathway Registry

**DOI:** 10.3389/fcvm.2023.1227217

**Published:** 2023-08-14

**Authors:** Liesbeth Rosseel, Darren Mylotte, Bernard Cosyns, Maarten Vanhaverbeke, David Zweiker, Rui Campante Teles, Oskar Angerås, Antoinette Neylon, Tanja Katharina Rudolph, Joanna J. Wykrzykowska, Tiffany Patterson, Giulia Costa, Soledad Ojeda, Apostolos Tzikas, Marcel Abras, Lionel Leroux, Eric Van Belle, Didier Tchétché, Sabine Bleiziffer, Martin J. Swaans, Radoslaw Parma, Daniel J. Blackman, Nicolas M. Van Mieghem, Marek Grygier, Simon Redwood, Bernard Prendergast, Guy Van Camp, Ole De Backer

**Affiliations:** ^1^ Department of Cardiology, Algemeen Stedelijk Hospital, Aalst, Belgium; ^2^ Faculteit Geneeskunde, Vrije Universiteit Brussel (VUB), Brussels, Belgium; ^3^ Department of Cardiology, University Hospital Galway and National University of Ireland, Galway, Ireland; ^4^ Centrum Voor Hart- en Vaatziekten (CHVZ), Universitair Ziekenhuis Brussel (UZ Brussel), Brussels, Belgium; ^5^ Department of Cardiology, AZ Delta, Roeselare, Belgium; ^6^ Division of Cardiology, Department of Cardiology and Intensive Care, Clinic Ottakring, Medical University of Graz, Graz, Austria; ^7^ Centro Hospitalar de Lisboa Ocidental (CHLO), Hospital de Santa Cruz; ^8^ Nova Medical School, Centro de Estudo de Doenças Crónicas (CEDOC), Lisbon, Portugal; ^9^ Department of Cardiology, Sahlgrenska University Hospital, Gothenberg, Sweden; ^10^ Department of Molecular and Clinical Medicine, Institute of Medicine, Gothenburg University, Gothenburg, Sweden; ^11^ Institut Cardiovasculaire Paris Sud, Ramsay Santé, Massy, France; ^12^ Clinic of General and Interventional Cardiology, Heart and Diabetes Center Nordrhine Westfalia, Ruhr-University, Bad Oeynhausen, Germany; ^13^ Department of Cardiology, University Medical Center Groningen, Groningen, Netherlands; ^14^ Department of Cardiology, Guys and St Thomas’ NHS Foundation Trust London, London, United Kingdom; ^15^ Cardiac Catheterization Division, Cardiothoracic and Vascular Department, Azienda Ospedaliero-Universitaria Pisana, Pisa, Italy; ^16^ Division of Interventional Cardiology, Reina Sofia Hospital, Maimonides Institute for Research in Biomedicine of Córdoba (IMIBIV), University of Córdoba, Córdoba, Spain; ^17^ Department of Cardiology, European Interbalkan Medical Centre, Thessaloniki, Greece; ^18^ University Clinic of Interventional Cardiology, Nicolae Testemitanu State University of Medicine and Pharmacy from Republic of Moldova, Chişinău, Moldova; ^19^ Medico-Surgical Department of Valvulopathies, CHU De Bordaux, Pessac, France; ^20^ CHU Lille, Institut Cœur Poumon, Pôle Cardiovasculaire et Pulmonaire, ACTION Group, Inserm U1011, Institut Pasteur de Lille, EGID, Université de Lille, Lille, France; ^21^ Department of Interventional Cardiology, Clinique Pasteur, Toulouse, France; ^22^ Department of Thoracic and Cardiovascular Surgery, Heart and Diabetes Center North Rhine-Westphalia, Bad Oeynhausen, Germany; ^23^ Department of Cardiology, St. Antonius Hospital, Nieuwegein, Netherlands; ^24^ Department of Cardiology and Structural Heart Diseases, 3 Division of Cardiology, Medical University of Silesia, Katowice, Poland; ^25^ Department of Cardiology, Leeds Teaching Hospitals, Leeds, United Kingdom; ^26^ Department of Interventional Cardiology, Erasmus University Medical Center, Rotterdam, Netherlands; ^27^ Chair and 1st Department of Cardiology, Poznan University of Medical Sciences, Poznan, Poland; ^28^ Department of Cardiology, Heart Center OLV Aalst, Aalst, Belgium; ^29^ Heart Center, Rigshospitalet, Copenhagen, Denmark

**Keywords:** Transcatheter aortic valve implantation, aortic stenosis, multidisciplinary Heart Team, minimalist TAVI, early discharge

## Abstract

**Background:**

A steep rise in the use of transcatheter aortic valve implantation (TAVI) for the management of symptomatic severe aortic stenosis occurred. Minimalist TAVI procedures and streamlined patient pathways within experienced Heart Valve Centres are designed to overcome the challenges of ever-increasing procedural volume.

**Aims:**

The 2022 European TAVI Pathway Survey aims to describe contemporary TAVI practice across Europe.

**Materials and methods:**

Between October and December 2022, TAVI operators from 32 European countries were invited to complete an online questionnaire regarding their current practice.

**Results:**

Responses were available from 147 TAVI centres in 26 countries. In 2021, the participating centres performed a total number of 27,223 TAVI procedures, with a mean of 185 TAVI cases per centre (median 138; IQR 77–194). Treatment strategies are usually (87%) discussed at a dedicated Heart Team meeting. Transfemoral TAVI is performed with local anaesthesia only (33%), with associated conscious sedation (60%), or under general anaesthesia (7%). Primary vascular access is percutaneous transfemoral (99%) with secondary radial access (52%). After uncomplicated TAVI, patients are transferred to a high-, medium-, or low-care unit in 28%, 52%, and 20% of cases, respectively. Time to discharge is day 1 (12%), day 2 (31%), day 3 (29%), or day 4 or more (28%).

**Conclusion:**

Reported adoption of minimalist TAVI techniques is common among European TAVI centres, but rates of next-day discharge remain low. This survey highlights the significant progress made in refining TAVI treatment and pathways in recent years and identifies possible areas for further improvement.

## Introduction

Transcatheter aortic valve implantation (TAVI) now has a class IA indication for the treatment of symptomatic severe aortic stenosis in patients aged 75 years or more, regardless of surgical risk (1). Since the first-in-human TAVI was performed in 2002, procedural refinement achieved through successive design iterations, improved pre-procedural planning, and optimised implant technique have resulted in improved outcomes and rapid expansion of the technique. TAVI procedures now outnumber surgical aortic valve replacement (SAVR) in many countries, and the forecast is a further increase of its application due to population ageing and expanding procedural indications (2).

To preserve safety, efficiency and patient outcomes alongside accelerating TAVI demand, optimisation of TAVI patient pathways, and minimalist TAVI procedures are essential to balance the burden on healthcare systems and resources. There are no data available to define how TAVI is currently organised and performed across Europe and whether recommendations and guidelines are incorporated into a daily practice. The 2022 European TAVI Pathway Survey was set up to provide insights as to how contemporary TAVI pathways and procedures are organised and identify potential areas of improvement that may further improve healthcare impact.

## Materials and methods

### Study design

The 2022 European TAVI Pathway Survey is a non-funded, international, multi-centre, observational, transverse study. A list of TAVI centres in 32 European countries was generated based upon information provided by national cardiac societies, national registries, and additional PubMed searches. TAVI operators for each centre identified through the investigators’ network and PubMed searches were invited by email to participate. The questionnaire was set up on an electronic web-based platform (SurveyMonkey) and distributed via digital link. Participation was voluntary and anonymous. The questionnaire was requested to be completed by a TAVI operator. The survey was open between 1 October and 15 December 2022, and a second wave to further improve participation was open between 1 and 10 January 2023.

The survey consisted of 35 (mostly) multiple-choice questions and was compiled based on the specific requirements for a Heart Valve Centre defined in the 2021 European guidelines on valvular heart disease (Supplementary Table S1) (1). The questionnaire (Supplementary Table S2) was categorised into four main areas: (1) patient selection, (2) pre-procedural work-up, (3) TAVI procedure, and (4) patient flow/pathway. The participants were asked to submit their answers based on current TAVI practice in their centre for “regular” elective transfemoral TAVI cases. Only surveys with ≥85% completion and including a response to the question concerning country of origin and number of TAVI procedures performed in 2021 were included in analyses.

### Definitions

Results were analysed overall and according to geographic region and centre procedural volume. Six geographic regions were defined: (1) DACH [Germany (D), Austria (A), Switzerland (CH)], (2) Nordic (Denmark, Finland, Iceland, Norway, Sweden), (3) BeNeFrance (Belgium, France, Luxembourg, the Netherlands), (4) UK/IRL (United Kingdom/ Republic of Ireland), (5) Southern Europe (Cyprus, Greece, Italy, Malta, Portugal, Spain), and (6) Eastern Europe (Albania, Bulgaria, Bosnia Herzegovina, Croatia, Czech Republic, Estonia, Hungary, Kosovo, Latvia, Lithuania, Montenegro, North Macedonia, Poland, Republic of Moldova, Romania, Serbia, Slovakia, Slovenia). Centre volume was defined by the number of TAVI cases performed in 2021 and divided into five categories: (1) <50 cases, (2) 50–99 cases, (3) 100–199 cases, (4) 200–499 cases, and (5) ≥500 cases. The number of TAVI cases per operator was calculated by dividing the number of TAVI cases by the number of TAVI operators in each centre and was not adjusted per local practice (e.g., one vs. two independent TAVI operators per case).

Alternative vascular access was reported as five separate approaches, each with two sub-categories: (1) transfemoral (balloon- or lithotripsy-assisted access), (2) transaxillary (direct percutaneous or surgical cutdown), (3) transthoracic (transapical or direct aortic approach), (4) transcarotid (direct percutaneous or surgical cutdown), and (5) transvenous (transcaval or transseptal approach).

Post-procedure transfer location was classified into high-care (intensive care unit), medium-care (recovery room, cardiac care unit, mid-care unit), and low-care (cardiology ward) unit.

### Statistical analysis

Descriptive statistics were used to present the data. Continuous variables are reported as mean and standard deviation (±SD) in a normal number distribution and as median and interquartile range (IQR) for skewed number distribution. Categorical variables are presented as percentages.

### Enrolment

In total, 688 European TAVI centres—with corresponding contact—were identified. One hundred and fifty-six centres (23%) responded to the survey: complete responses were available for 147 (94%) centres and 9 (6%) submitted partial results. Overall completion rate of the questionnaire was 94%, with an average time of 8 min and 24 s required. An overview of enrolment per country is provided in Supplementary Table S3.

## Results

### Centres

**Figure 1:**
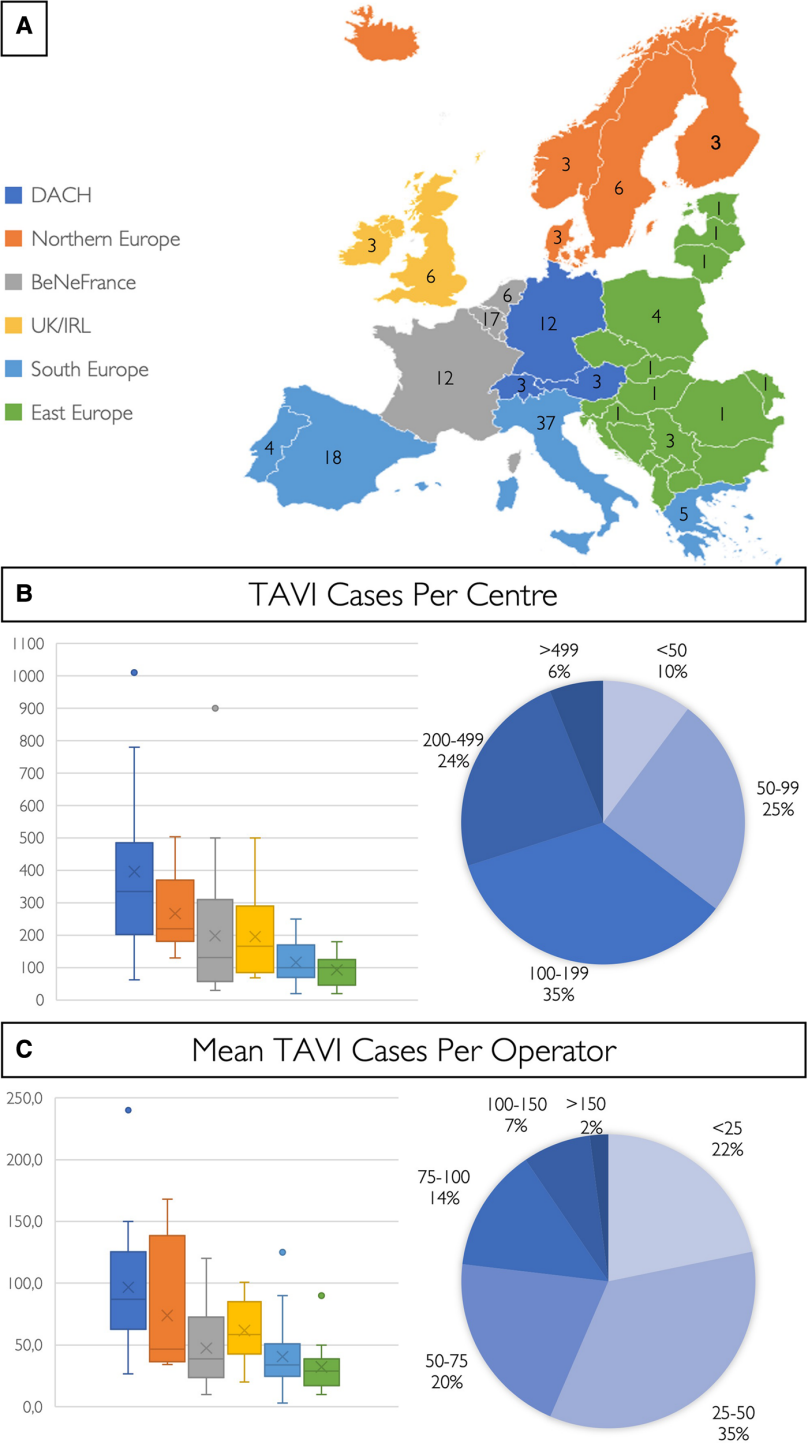
European TAVI pathway survey. (**A**) Colour coding for the six different European regions and number of centres of each country included in this study. The number of TAVI cases per centre (**B**) and the mean number of TAVI cases per operator (**C**) are shown in a box and whisker plot for the six different European regions, and cake diagrams showing categorised case and mean operator volumes [BeNeFrance, Belgium, France, Luxemburg, the Netherlands; DACH, Germany (D), Austria (A), Switzerland (CH); and UK/IRL, United Kingdom/Republic of Ireland].

The 147 participating centres in 26 different countries performed a total of 27,223 TAVI cases in 2021 (Figure 1). Three quarters of respondents work in public hospitals (76%, *n* = 112), 14% (*n* = 21) in a private setting, and 10% (*n* = 14) in mixed public/private practice. The average number of TAVI cases per centre was 185 (median 138; IQR 77–194), the largest centre performing 1,010 cases and the smallest 20 cases (Figure 1). In 2021, the centres with the highest TAVI volume were situated in the DACH region (median 335; IQR 219–490), followed by the Nordic region (median 220; IQR 200–350), while the regions with the lowest number of TAVI cases per centre were in Southern (median 101; IQR 70–169) and Eastern Europe (median 100; IQR 53–122) (Supplementary Table S4). The average number of TAVI cases per operator was 52 (median 43; IQR 26–69) (Figure 1; Supplementary Table S5), and in 22% (*n* = 32) of participating centres, the mean number of TAVI cases per operator was <25 per annum.

### Treatment strategy

**Figure 2:**
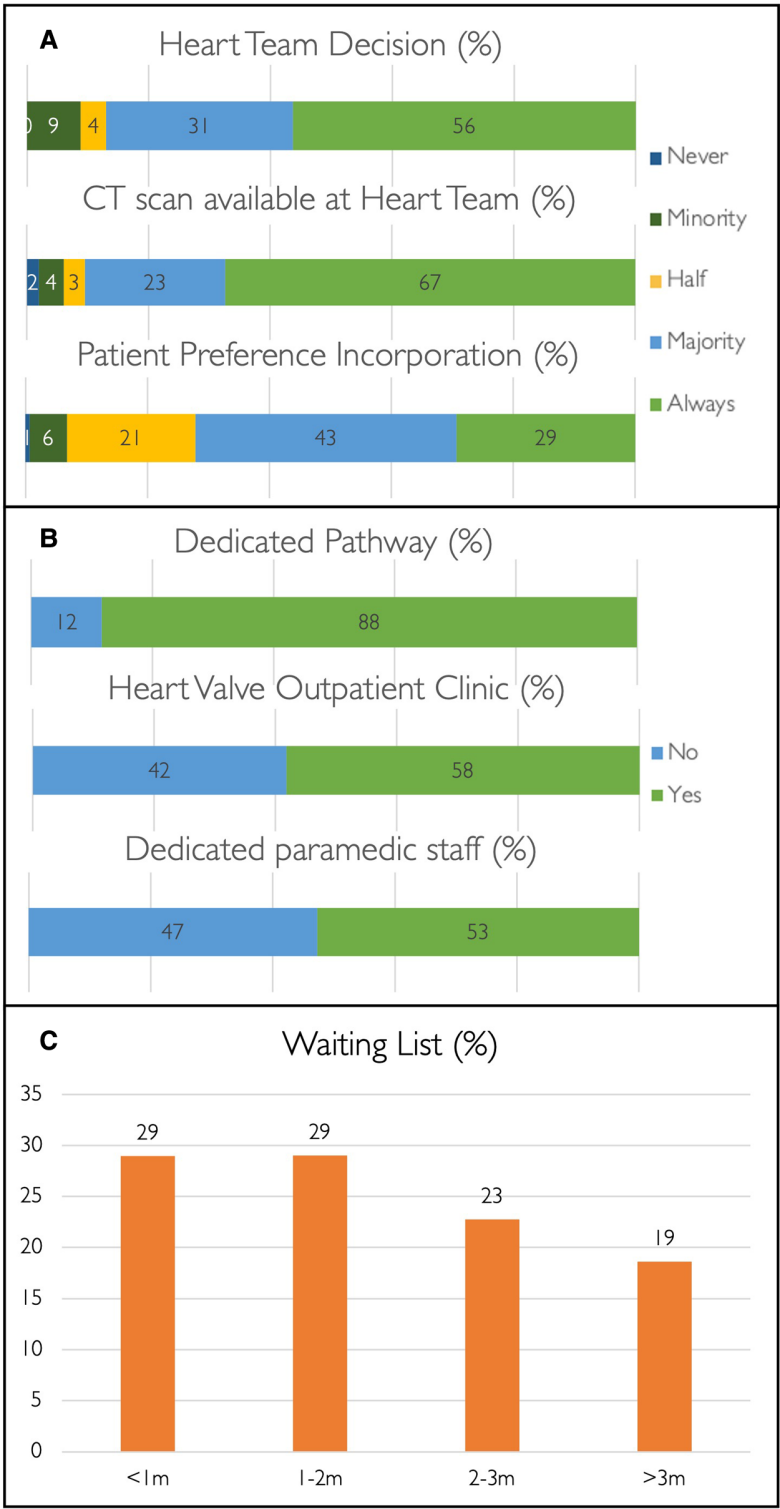
TAVI pathway. Bar charts and percentages demonstrating TAVI decision-making (**A**) and patient pathways (**B**), and TAVI waiting times (**C**).

Treatment strategy is guided by the Heart Team in 87% of centres (Figure 2) where a multi-slice computed tomography (MSCT) scan is available to assist decision-making in 90% of cases. Patient preference is considered in 72% of case discussions (Figure 2). Among patients with isolated severe aortic stenosis and favourable transfemoral access, 45% (*n* = 66) and 41% (*n* = 60) of operators use the ages of 75 and 80 years, respectively, as the threshold for TAVI as the default treatment strategy. In 1% (*n* = 1) and 5% (*n* = 8), the ages of 65 and 70 years are used as the threshold for TAVI as the preferred treatment strategy, while the remainder (6%, *n* = 9) indicated TAVI as the first choice in patients aged 85 years or older.

### Pathway

A structured TAVI pathway is present in 88% of centres (*n* = 129); 58% (*n* = 85) have a dedicated specialist-led outpatient heart valve clinic, and 53% (*n* = 77) have para-medical support staff (e.g., clinical nurse specialist or non-clinical coordinator) available (Figure 2). Most centres have waiting times of ≤3 months for TAVI (81%, *n* = 118), and this is even below 2 weeks in 10% (*n* = 14) but above 6 months in 5% (*n* = 7) (Figure 2). Waiting times of >3 months are observed across the spectrum of centre volume and appear irrespective of the availability of a structured pathway, a heart valve outpatient clinic, or presence of para-medical staff. DACH is the only region without a TAVI waiting time of >3 months (Supplementary Table S6).

### Pre-procedural work-up

**Figure 3:**
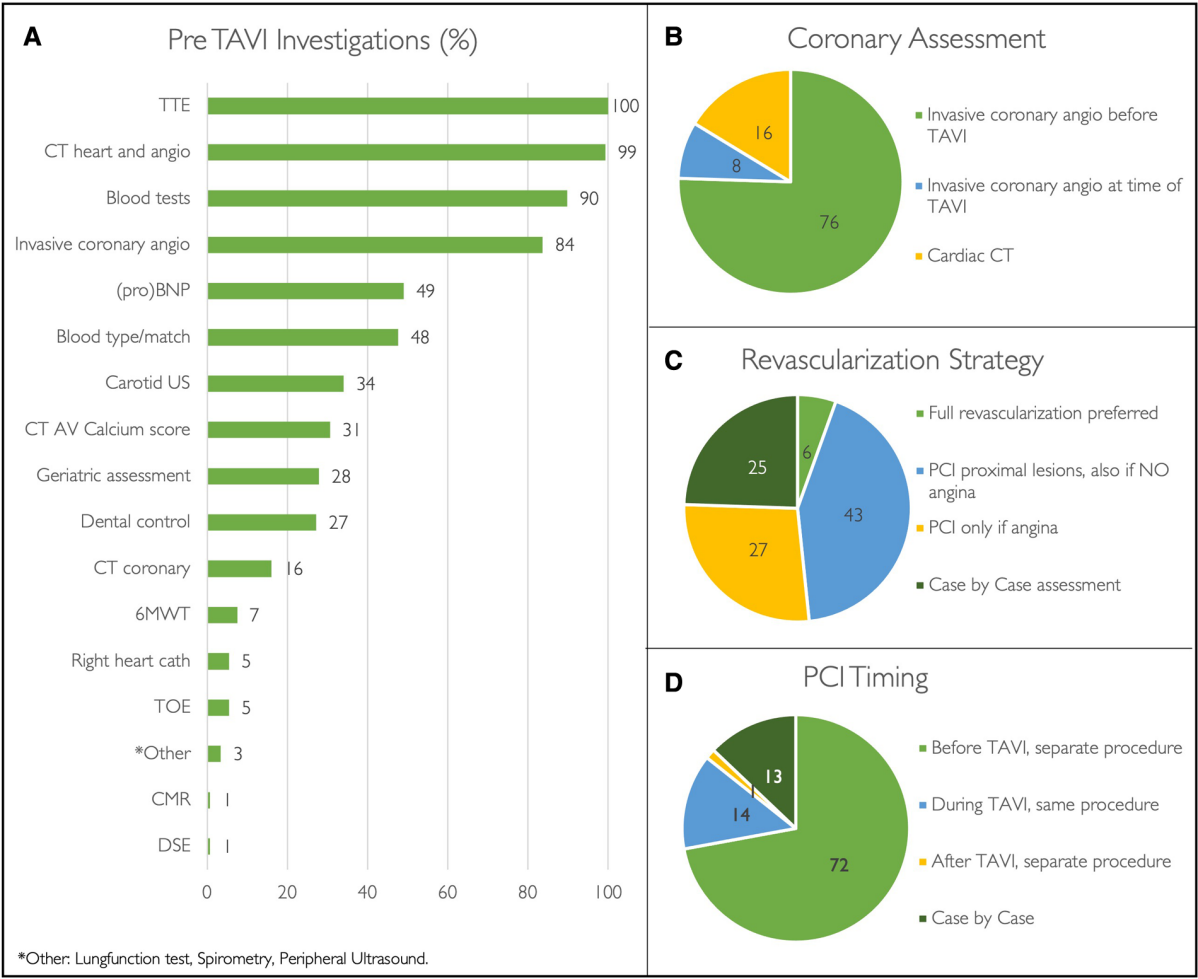
Pre-TAVI investigations. Pre-procedural investigations performed during routine TAVI work-up (**A**). Pie diagrams demonstrating coronary revascularization strategies (**B-D**) expressed as percentages. (Angio, angiogram; AV, aortic valve; CMR, cardiac magnetic resonance scan; CT, Computed tomography; DSE, dobutamine stress echocardiography; PCI, percutaneous coronary intervention; proBNP, N-terminal pro B-type natriuretic peptide; TOE, transoesophageal echocardiography; TTE transthoracic coronary echocardiography; US, ultrasound; 6MWT, six-minute walking test).

Transthoracic echocardiography (TTE), MSCT plus angiogram, and blood tests are the standard of care in the majority of centres (Figure 3). These pre-procedural investigations are performed either during hospital admission (25%, *n* = 36) or during one, two, or multiple separate outpatient visits in 20% (*n* = 29), 16% (*n* = 24), and 37% (*n* = 54) of institutions, respectively. The pre-TAVI MSCT is analysed by a TAVI operator in 60% (*n* = 88), a clinical specialist from a MedTech company in 20% (*n* = 30), a radiologist in 12% (*n* = 18), or a trained fellow/nurse in 3% (*n* = 5).

All surveyed centres perform coronary work-up prior to TAVI, either with invasive coronary angiography (84%, *n* = 123) or by means of the pre-TAVI MSCT (16%, *n* = 24) (Figure 2). Among patients with significant coronary artery disease that are accepted for TAVI, revascularisation strategy and timing of percutaneous coronary intervention (PCI) widely differ (Figure 2). When referring hospitals have a catheter laboratory onsite (87%, *n* = 128), PCI is performed in the referring centre in 23% (*n* = 30), in the TAVI centre in 17% (*n* = 22), and variably in either the TAVI centre or referring centre in the remaining 60% (*N* = 76) according to case complexity.

### TAVI procedure

**Figure 4:**
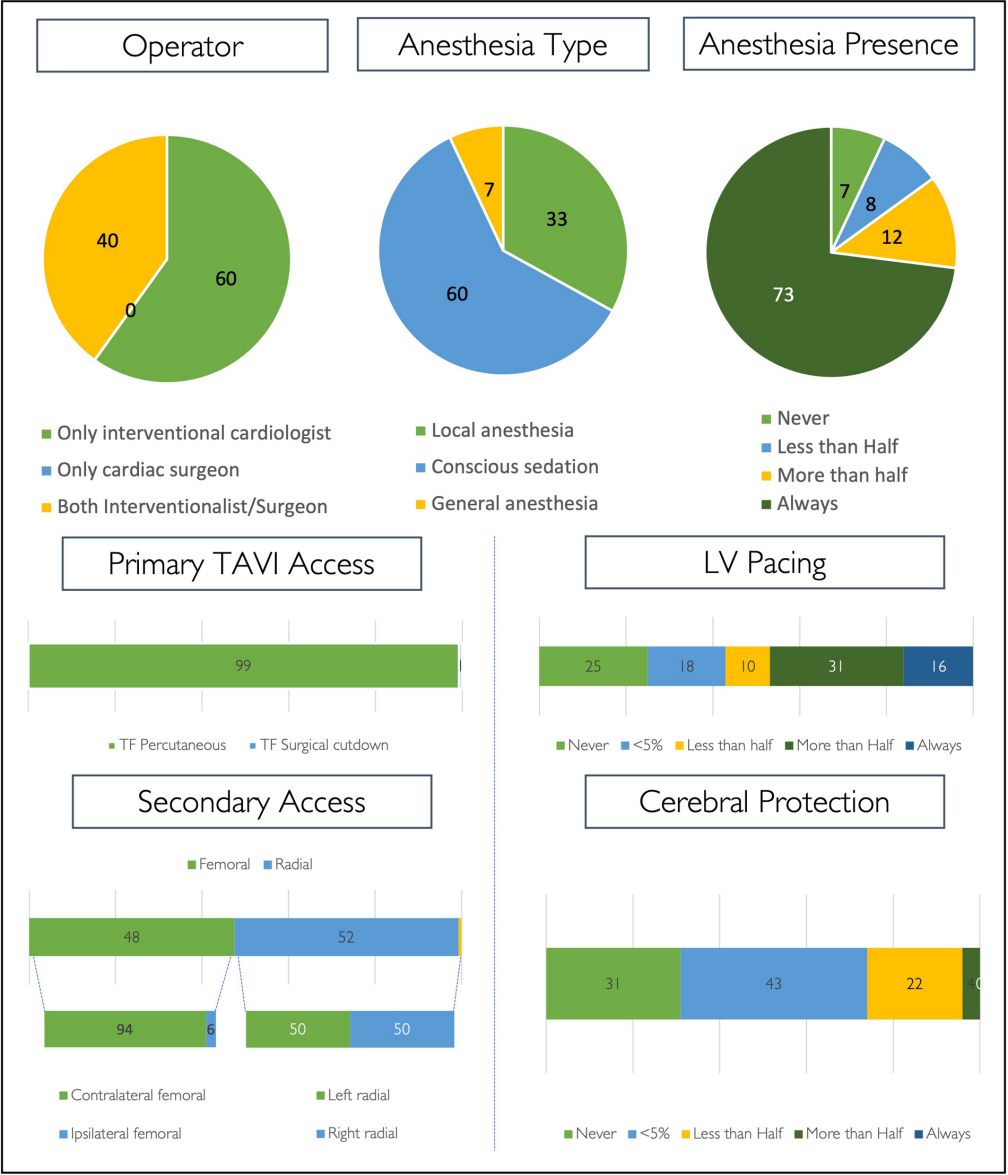
TAVI procedure. Procedural strategy for routine transfemoral TAVI cases, expressed as percentages. (LV, left ventricle).

TAVI procedures are performed in most cases by interventional cardiologist teams only (60%), and the remainder are performed by mixed teams of interventionalists and cardiac surgeons (Figure 4).

Across all centres, local anaesthesia with conscious sedation is the most widely adopted anaesthetic strategy (60%) for routine TAVI. Local anaesthesia without conscious sedation is the default strategy in 33% (*n* = 49) of centres and most infrequently used in centres performing <50 cases per annum (7%). General anaesthesia is used in the minority of centres (7%, *n* = 10), is less implemented for regular TAVI cases in centres performing >100 cases per annum (4%, *n* = 4) as compared with centres performing <100 cases (12%, *n* = 6), and is only observed in the Southern European and BeNeFrance regions (Figure 4, Supplementary Table S7). Overall, an anaesthetic team is always present in the catheter laboratory during regular transfemoral TAVI in 73% of centres (*n* = 107). Further details on procedural strategy are shown in Figure 4.

Participants were asked to indicate alternative access sites employed in a sequence of preference/performance (Supplementary Table S8). Percutaneous transluminal angioplasty (PTA)-assisted transfemoral access—either with a plain or intravascular lithotripsy balloon—is the first choice for alternative access in 67% (78% plain balloon; 22% lithotripsy) and the second choice in 58% (28% plain balloon; 72% lithotripsy). The most preferred alternative access site as a third option is transaxillary (50%), more often using surgical cutdown (65%) as compared with a direct percutaneous approach (35%). Transapical access is chosen in 6%, 9%, and 12% as a first, second, and third choice for alternative access, respectively. When using the transcarotid approach, surgical cutdown is most often preferred (94%). Transvenous access is the least preferred approach for alternative access—the majority is transcaval (69%).

### Post-procedural patient flow

**Figure 5:**
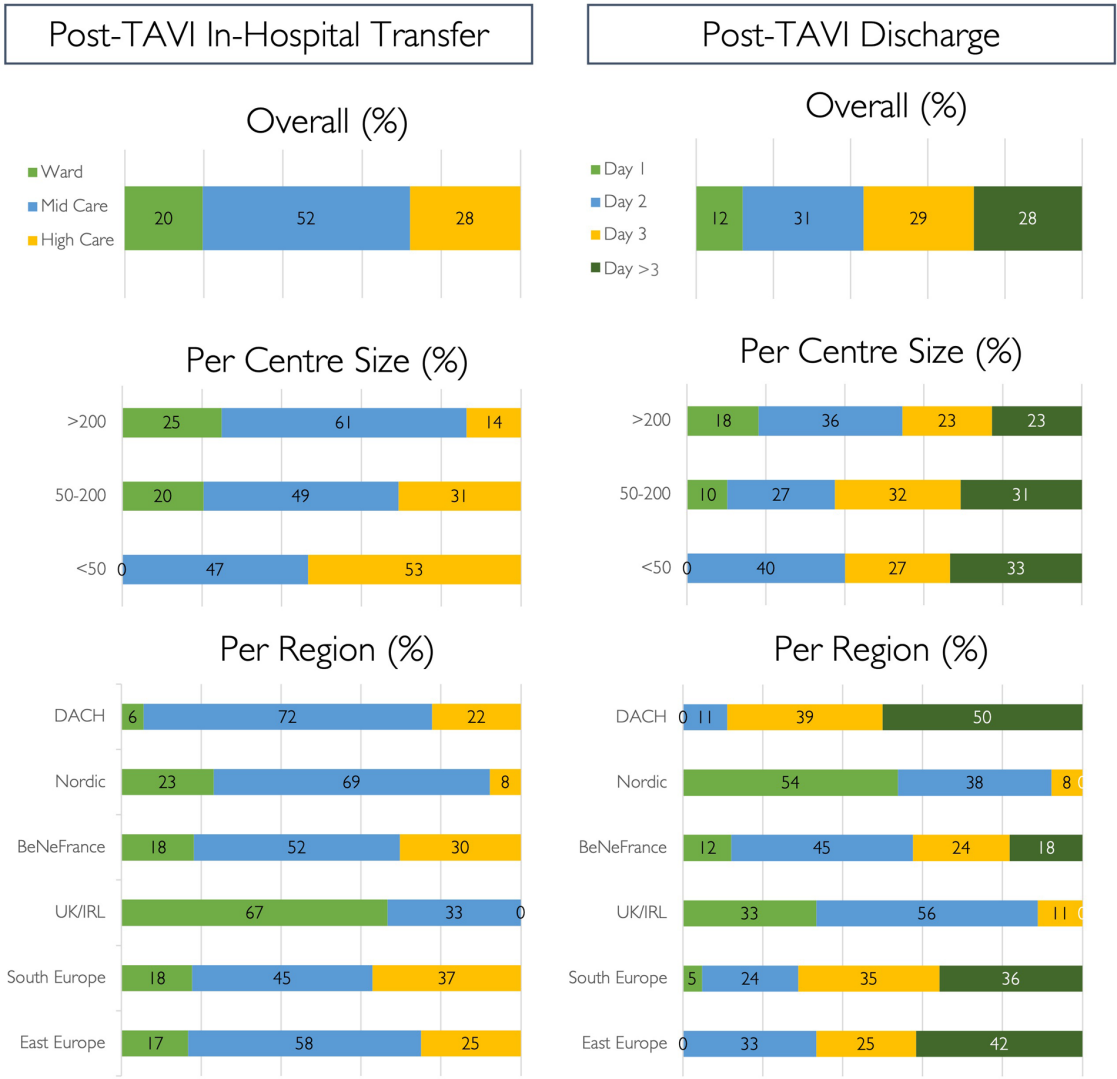
Post-TAVI strategy. Post-procedural strategy for in-hospital step down and discharge following routine uncomplicated transfemoral TAVI cases, expressed as percentages.

After an uncomplicated transfemoral TAVI procedure, patients are transferred to a low-care facility (28%, *n* = 29) and discharged from the hospital on post-procedural day 1 (12%, *n* = 17) (Figure 5). Same-day discharge is never carried out, but 25% (*N* = 38) of participants would consider this strategy in selected cases. Results for in-hospital transfer and discharge timing according to region and centre size are shown in Figure 5.

### Registration/teaching

Most centres (92%, *n* = 132) record cases in a TAVI database at the institutional level, while only 74% (*n* = 99) and 12% (*n* = 11) register in national or international databases, respectively. Educational meetings (e.g., for medical staff, nurses, general practitioners, etc.) are organised on a regular basis in 67% (*n* = 98) of centres. In total, 84% (*n* = 122) of participants participate in research or clinical trials.

## Discussion

The 2022 European TAVI Pathway Survey provides important insights as to how contemporary TAVI procedures and pathways are organised and executed across the continent. Data from 147 centres in 26 European countries were collected, and the salient observations were (1) guideline-directed Heart Team decision-making has been widely adopted, but patient preferences are not commonly incorporated in this discussion; (2) one-third of centres (35%) perform <100 TAVI cases per year; (3) most centres have a structured TAVI patient pathway, but only half have a dedicated heart valve clinic supported by para-medical staff; (4) although waiting times for TAVI are acceptable, one in five centres have a waiting list of >3 months; (5) the vast majority of cases are performed without general anaesthesia, but an anaesthetic team is still present in most cases (73%); and (6) post-procedure transfer to a low-care unit and next-day discharge after uncomplicated TAVI rarely occur.

A sharp increase in TAVI procedural volume is predicted within the next decades due to expanding indications and an ageing population (2, 3). TAVI consumes less healthcare resources than SAVR, mainly driven by shorter hospital stay, less post-procedural rehabilitation, and fewer short- and long-term complications (4). To pursue and sustain these advantages of TAVI in the face of ever-increasing demand, streamlined patient pathways and minimalistic procedures need to be incorporated into a daily practice. The current study reports how TAVI centres are organised across Europe for the first time and provides important insight concerning potential areas of improvement within current TAVI processes.

Multidisciplinary Heart Team-based decisions increase the application of guideline recommendations and subsequently improve patient outcomes (1). This survey demonstrates that Heart Team decisions are widely applied within the TAVI population (87% of centres) and that a MSCT scan is usually available to inform this discussion (90% of centres). Information gained from a pre-TAVI MSCT scan is essential for detailed risk stratification and procedural planning (SAVR vs. TAVI), reflecting the 2021 European Guidelines on valvular heart disease (1). Interestingly, 16% of centres consider the pre-procedural MSCT as an alternative to the invasive assessment of coronary artery disease. Expansion of this strategy has the potential to further simplify the TAVI pathway but requires further supportive evidence (5).

A patient-centred approach to treatment decisions is axiomatic in the setting of severe aortic stenosis and an important tenet of the 2021 European guidelines on valvular heart disease (1). Despite this guidance, patient preference does not appear to have a sufficient weight in Heart Team discussions. This finding suggests that the cardiovascular community should make greater efforts to more consistently implement this guideline recommendation, ideally within the setting of a dedicated heart valve clinic which provides the perfect opportunity to inform a patient (and caregiver) of potential treatment choices and subsequently discuss informed patient preferences.

Access to heart valve clinics improves adherence to guidelines and enhances detection of disease progression to ensure optimal timing of surgical or transcatheter intervention which is associated with improved patient outcomes (6–8). Heart valve clinics are essential to coordinate care, standardise patient flow, and limit TAVI waiting times. This study demonstrates that structured patient pathways are widely adopted (88%), but only half of the responding centres have a dedicated heart valve clinic supported by dedicated para-medical staff. Although the lack of specialised nurses or non-clinical coordinators is often financially driven, we hypothesise that their involvement provides a beneficial impact on efficiency and cost-effectiveness, as observed in heart failure programmes (9, 10). Potential areas of focus include the avoidance of futile or repeated pre-procedural tests, timely detection of disease progression, waiting list management, matching devices to Heart Team recommendation, and improving early discharge or access to stepdown care. While these assumptions have yet to be investigated, we believe that the role of dedicated heart valve clinics and para-medical staff will become even more apparent as global TAVI volumes increase.

Longer TAVI waiting times are associated with worse pre- and post-procedural outcomes (11, 12) and exceeded 3 months in nearly 20% of participating centres. This finding was observed in all categories of centre volume and organisation (but not in the DACH region) (Supplementary Table 6). These findings highlight the need for strategies to minimise delays in access to TAVI, especially in the face of increasing demand.

In 2019, 669 TAVI centres registered in the US TVT registry with an average procedure volume per site of 110 with a median of 84 (IQR 50–137) and 24% of centres performing <50 cases per annum were identified (13). In this contemporary European study, the average procedure volume per site was 185 with a median of 138 (IQR 77–194), with only 10% of centres performing <50 cases annually. Importantly, lower operator experience and volume have been associated with increased rates of adverse TAVI outcomes (14, 15). In the US consensus statements on advanced interventional cardiology training and the operator and institutional requirements for TAVI centres, a minimum of 50 cases per year is proposed for TAVI programmes, although recommendations concerning individual operator volumes are not specified (16, 17). The target of >50 cases per annum is reached in 90% of our study population. As with coronary interventions, European guidelines should inform policymakers of Heart Valve Centre requirements to avoid overgrowth of small, less-experienced centres.

A minimalist procedural approach should be applied to allow development of a streamlined TAVI programme. Local anaesthesia without the need for an anaesthetic team, secondary radial access, left ventricular pacing protocols, and early mobilisation have proven safety in multiple studies (18–24). Despite minimalist TAVI modifications being widely adopted, allowing faster post-procedural ambulation, this does not translate into greater post-procedural use of low-care facilities or faster discharge in this study. Indeed, uncomplicated TAVI patients are frequently transferred to medium- or high-care facilities (51% and 30%, respectively), and next-day discharge rates remain low (12%), with zero same-day discharge. Local protocols, politics, and/or legislation may influence these practices and unfortunately increase healthcare burden.

### Study limitations

Although we identified 688 TAVI centres in 32 countries, an official database of European TAVI centres is unavailable, and some centres may therefore have been overlooked in the invitation to participate in this survey. Selection bias is possible, favouring centres with an underlying interest in scientific research—underestimation of the number of smaller volume centres within this study population is therefore possible. The questionnaire related only to the operator's practice at the time the survey was completed. Data on TAVI volume and practice in earlier periods were not included, nor were data on surgical valve replacement. The procedural volume of individual operators may be higher than represented since this depends on the distribution of procedures in each centre. Questions related only to regular transfemoral TAVI cases and baseline clinical profiles may differ between centres and countries.

## Conclusion

The practice of TAVI across European centres varies widely. Heart Team decision-making, transfemoral access, and local anaesthesia are the norm. However, areas for improvement include greater account of individual patient preference into clinical decision-making, more frequent use of early discharge protocols, and continued attention to prolonged TAVI waiting times. These data identify areas for further refinement of TAVI pathways and procedures within the rapidly evolving European context.

## Impact on a daily practice

A steep rise in TAVI has evolved in recent years, but no studies investigated how TAVI procedures and pathways are currently organised across Europe. In the 2022 European TAVI Pathway Registry, we collected data from 147 TAVI centres in 26 different European countries, including low- to high-volume centres. Adoption of minimalist TAVI techniques and guideline-directed Heart Team decision-making is common among European TAVI centres, but rates of next-day discharge and incorporation of patient’s preferences in the decision-making process remain low. The results highlight the significant progress made in refining TAVI treatment and pathways and their implementation but also identify possible areas for further improvement.

## Data Availability

The original contributions presented in the study are included in the article/Supplementary Material, further inquiries can be directed to the corresponding author.
