# Supplementary material for: Contemporary European practice in transcatheter aortic valve implantation: results from the 2022 European TAVI Pathway Registry
Source: Front Cardiovasc Med. 2023 Aug 14;10:1227217. doi: 10.3389/fcvm.2023.1227217 (PMC10461475; doi:10.3389/fcvm.2023.1227217)
Supplement: Supplementary file 1 [file Table1.docx]

**Supplemental Table 1.** Requirements for a Heart Valve Centre.

| Centre performing heart valve procedures with institutional cardiology and cardiac surgery departments. |
| --- |
| **Heart** **Team**: Clinical cardiologist, interventional cardiologist, cardiac surgeon, imaging specialist with expertise in interventional imaging, cardiovascular anaesthesiologist.  **Additional specialists if required**: heart failure specialist, electrophysiologist, geriatrician and other specialists (intensive care, vascular surgery, infectious disease, neurology). Dedicated nursing personnel is an important asset to the Heart Team.  The Heart Team must meet on a frequent basis and work with standard operating procedures and clinical governance arrangements defined locally.  A hybrid catheterization laboratory is desirable.  The entire spectrum of surgical and transcatheter valve procedures should be available.  High volume for hospital and individual operators. |
| Multimodality imaging including echocardiography, CCT, CMR, and nuclear medicine, as well as expertise on guidance of surgical and interventional procedures. |
| Heart Valve clinic for outpatient and follow-up management. |
| Data review: continuous evaluation of outcomes with quality review and/or local/external audit.  Education programmes targeting patient primary care, operator, diagnostic and interventional imager training and referring cardiologist. |

CCT, cardiac computed tomography; CMR, cardiac magnetic resonance.
