# Supplementary material for: Contemporary European practice in transcatheter aortic valve implantation: results from the 2022 European TAVI Pathway Registry
Source: Front Cardiovasc Med. 2023 Aug 14;10:1227217. doi: 10.3389/fcvm.2023.1227217 (PMC10461475; doi:10.3389/fcvm.2023.1227217)
Supplement: Supplementary file 2 [file Table2.docx]

**Supplemental Table 2.** Survey Questions.

| 1 | Country *(mandatory)* | 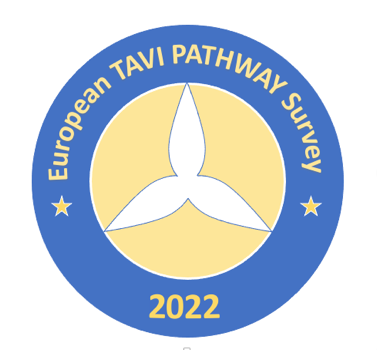 |
| --- | --- | --- |
| 2 | Health Care System *(mandatory)* | - Public - Private - Mixed Public and Private - Other |
| **TAVI Patient selection** | | |
| 3 | Treatment strategy (TAVI vs SAVR) in patients with aortic valve disease is discussed in a ´Heart Team´ meeting (i.e., shared decision in a multidisciplinary team discussion where at least a cardiologist and cardiac surgeon are present) | - Always - Majority of cases - About half of the cases - Minority of cases - Never |
| 4 | Pre-procedural cardiac/vessel CT scan is available for the Heart Team discussion. | - Always - Majority of cases - About half of the cases - Minority of cases - Never |
| 5 | “In the Heart team meeting, some patients are nearly directly accepted for TAVI once the diagnosis of severe AS is agreed.” This is true for patients with isolated severe AS and good transfemoral access. | - Agree, in patients >65 years old - Agree, in patients ≥70 years old - Agree, in patients ≥75 years old - Agree, in patients ≥80 years old - Agree, in patients ≥85 years old - Disagree |
| 6 | Expectations and preferences of the informed patient are actively incorporated in the Heart Team discussion (i.e., ´Shared decision-making process´). | - Always - Usually - Sometimes - Rarely - Never |
| **PRE TAVI Procedure** | | |
| 7 | Which investigations are always performed during a ´regular´ pre-TAVI work-up (´Screening phase´) (More than 1 choice is possible) | - Transthoracic echocardiography (TTE) - Transoesophageal echocardiography (TOE) - Dobutamine stress echocardiography (DSE) - CT calcium score aortic valve - CT heart + CT Angio for access - CT coronary - Invasive coronary angiogram - Right heart catheterization - Cardiac magnetic resonance imaging (CMR) - Geriatric assessment: frailty assessment, mini-mental state exam, etc - 6-minute walking test - Dental control - Blood tests: Creatinine, complete blood count, etc - (NT-pro) BNP - Blood type and cross match - Carotid artery ultrasound - Other (please specify0 |
| 8 | In an elective TAVI patient, the pre-procedure work-up is done during: | - In Hosp admissions >1 day - One-stop visit (e.g., day ward) - Two-stop visit (e.g., day ward) - Separate out-patient visits - Other (please specify9 |
| 9 | The majority of pre-TAVI CTs is analysed by: | - clinical specialist of a MedTech company - Radiologist - TAVI operator - Trained fellow or nurse specialist - Other (please specify) |
| 10 | Strategy for the assessment of the coronary arteries in majority of patients referred/accepted for TAVI: | - An invasive coronary angiogram is performed before the TAVI procedure - An invasive coronary angiogram is performed at the same time of the TAVI procedure - Cardiac CT for coronary assessment is acceptable in some patients - Cardiac CT for coronary assessment is first choice diagnostic tool - Coronary assessment is not standardly performed - Other (please specify) |
| 11 | In patients with significant coronary artery disease and accepted for TAVI, strategy for coronary revascularization of significant lesions is: | - Full coronary revascularization is preferable for all cases - PCI only of proximal lesions, only if angina - PCI only of proximal lesions, also if no angina - PCI proximal to distal lesions, only if angina - PCI proximal to distal lesions, also if no angina - Never revascularization - Highly dependent on clinical, coronary and valvular characteristics - Other (please specify) |
| 12 | In patients accepted for TAVI with decision to perform coronary revascularization, PCI would be done in most cases: | - Before TAVI, in a separate procedure - During TAVI procedure, but before valve deployment - During TAVI procedure, but after valve deployment - After TAVI, in a separate procedure - Strategy highly dependent on clinical, coronary and valvular characteristics - Other (please specify) |
| 13 | When working with referring cardiology centres, and PCI is considered necessary: | - PCI always in the referring centre - PCI always in the TAVI centre - Depending on the case, PCI in referring or TAVI centre - Referring centres do not have a cathlab - We do not have referring centres - Other (please specify) |
| **TAVI Procedure** | | |
| 14 | How many TAVI procedures were performed in your centre in 2021? (Insert a whole number) *This field is mandatory |  |
| 15 | How many independent TAVI operators are there in your centre? |  |
| 16 | The TAVI operators are: | - Only interventional cardiologist - Only cardiac surgeons - Both interventional cardiologists and cardiac surgeons - Other (please specify) |
| 17 | In a standard transfemoral TAVI procedure, most TAVI cases are done in: | - General anaesthesia - Conscious sedation - Local anaesthesia - Other (please specify) |
| 18 | In a standard transfemoral TAVI procedure, an anaesthesiologist is present in the cathlab during TAVI: | - 100% - 75-99% - 50-75% - 25-50% - 3-25% - <5% - Never |
| 19 | In a standard transfemoral TAVI procedure, the primary access for TAVI is: | - Percutaneous transfemoral - Surgical cutdown transfemoral - Other (please specify) |
| 20 | In a standard transfemoral TAVI procedure, the usual secondary access for the pigtail catheter is: | - Contralateral femoral artery - Ipsilateral femoral artery - Left radial artery - Right radial artery - No secondary access, if judged feasible - Other (please specify) |
| 21 | Please rank alternative access TAVI approaches from 1 (first choice) to 10 (last choice), as used in your centre:(drag or change number, leave blank if technique not provided in your centre) | - Transfemoral, balloon angioplasty assisted - Transfemoral, lithotripsy (´shockwave´) assisted - Transaxillary, direct percutaneous - Subclavian, surgical cutdown - Transcaval - Transcarotid, direct percutaneous - Transcarotid, surgical cutdown - Transapical - Direct aortic - Transseptal |
| 22 | In a standard transfemoral TAVI procedure, pacing on the stiff LV wire (left ventricle) is used: | - 100% - 75-99% - 50-75% - 25-50% - 3-25% - <5% - Never |
| 23 | In a standard transfemoral TAVI procedure, a cerebral embolic protection device is used: | - 100% - 75-99% - 50-75% - 25-50% - 3-25% - <5% - Never |
| 24 | After a standard transfemoral uncomplicated TAVI procedure, the patient is post-procedure transferred to the: | - Intensive care unit (ICU) - Recovery Room - Cardiac care unit (CCU) - Mid care unit - Cardiology ward - Other (please specify) |
| 25 | After a standard transfemoral uncomplicated TAVI, the patient is discharged (on average) to home/residence on how many days post-procedure: | - Day 0 - Day 1 - Day 2 - Day 3 - Day 4-7 - After day 7 |
| 26 | “Same-day discharge TAVI can be considered in selected cases.” | - Strongly agree - Agree - Neither agree nor disagree - Disagree - Strongly disagree |
| **TAVI Patient Flow/Pathway** | | |
| 27 | Do you have a structured patient pathway for a TAVI patient in the pre-, peri- and post- procedural phase? | - Yes - No |
| 28 | Do you have a dedicated Heart Valve out-patient clinic at your centre? (i.e. Specialist led out-patient clinic for the management of heart valve disease patients) | - Yes - No |
| 29 | Do you have dedicated paramedic staff for the organization of TAVI patient flow e.g., clinical nurse specialist, non-clinical coordinator, etc.? | - Yes - No |
| 30 | The software or electronical platform currently used to manage the TAVI patient waiting list in your centre, is | - Shared excel/word file - A specific developed digital platform - A non-shared excel/word file - Waiting list is not electronical - A platform within the electronic patient system of the hospital - Other (please specify) |
| 31 | How long is the current waiting time (on average) for a standard elective transfemoral TAVI procedure in your centre? (i.e., time from diagnosis/indication for intervention by the clinician to TAVI procedure) | - < 2 weeks - 2-4 weeks - 1-2 months - 2-3 months - 3-6 months - 6-12 months - >12 months - Strongly dependent on public vs. private system |
| 32 | Is there a structured registration of TAVI procedures and outcomes in your centre? | - TAVI centre level: Yes/No - Regional level: Yes/No - National: Yes/No - International: Yes/No - Other (please specify) |
| 33 | Are there educational meetings on a regular basis for medical staff, nursing, general practitioners, etc.? | - Yes - No - Other (please specify) |
| 34 | Do you perform/participate in research or clinical trials in your TAVI practice? | - Yes - No - Other (please specify) |
| 35 | Any further comments regarding TAVI pathway processes? |  |
