# Supplementary material for: Contemporary European practice in transcatheter aortic valve implantation: results from the 2022 European TAVI Pathway Registry
Source: Front Cardiovasc Med. 2023 Aug 14;10:1227217. doi: 10.3389/fcvm.2023.1227217 (PMC10461475; doi:10.3389/fcvm.2023.1227217)
Supplement: Supplementary file 3 [file Table3.docx]

**Supplemental Table 3.** Enrolment per country.

|  | # TAVI centres with email address (%) | # Centres responded | Response Rate (%) | # Centres with Partial Response | # Centres with Complete response |
| --- | --- | --- | --- | --- | --- |
| Austria | 13 | 3 | 23 | 0 | 3 |
| Albania | NA |  |  |  |  |
| Belgium/Luxemburg | 24 | 17 | 71 | 0 | 17 |
| Bosnia Herzegovina | 1 | 0 | 0 | 0 | 0 |
| Bulgaria | 0 |  |  |  |  |
| Croatia | 2 | 1 | 50 | 0 | 1 |
| Czech Republic | 5 | 0 | 0 | 0 | 0 |
| Denmark | 4 | 3 | 75 | 0 | 3 |
| Estonia | 2 | 1 | 50 | 0 | 1 |
| Finland | 5 | 3 | 60 | 1 | 2 |
| France | 54 | 12 | 22 | 1 | 11 |
| Germany | 57 | 12 | 21 | 0 | 12 |
| Greece/Cyprus | 14 | 5 | 36 | 0 | 5 |
| Hungary | 4 | 1 | 25 | 0 | 1 |
| Iceland | 1 | 0 | 0 | 0 | 0 |
| Italy/Malta | 273 | 37 | 14 | 1 | 36 |
| Kosovo | NA |  |  |  |  |
| Latvia | 1 | 1 | 100 | 1 | 0 |
| Lithuania | 1 | 1 | 100 | 0 | 1 |
| Netherlands | 14 | 6 | 43 | 1 | 5 |
| Montenegro | NA |  |  |  |  |
| North Macedonia | NA |  |  |  |  |
| Norway | 5 | 3 | 60 | 0 | 3 |
| Poland | 22 | 4 | 18 | 1 | 3 |
| Portugal | 12 | 4 | 33 | 1 | 3 |
| Republic of Ireland | 6 | 3 | 50 | 0 | 3 |
| Republic of Moldova | 1 | 1 | 100 | 0 | 1 |
| Romania | 7 | 1 | 14 | 0 | 1 |
| Serbia | 3 | 3 | 100 | 1 | 2 |
| Slovakia | 1 | 1 | 100 | 0 | 1 |
| Slovenia | 1 | 0 | 0 | 0 | 0 |
| Spain | 64 | 18 | 28 | 0 | 18 |
| Sweden | 8 | 6 | 83 | 1 | 5 |
| Switzerland | 38 | 3 | 8 | 0 | 3 |
| United Kingdom | 34 | 6 | 18 | 0 | 6 |
| **TOTAL** | **688** | **156** | **23** | **9** | **147** |
