# Supplementary material for: Contemporary European practice in transcatheter aortic valve implantation: results from the 2022 European TAVI Pathway Registry
Source: Front Cardiovasc Med. 2023 Aug 14;10:1227217. doi: 10.3389/fcvm.2023.1227217 (PMC10461475; doi:10.3389/fcvm.2023.1227217)
Supplement: Supplementary file 4 [file Table4.docx]

**Supplemental Table 4**. TAVI cases performed in 2021 per region.

|  | TAVI Centres | TAVI Cases Total | Mean | ±SD | IQR1 | IQR3 | MEDIAN |
| --- | --- | --- | --- | --- | --- | --- | --- |
| DACH | 18 | 7132 | 396 | 246 | 219 | 490 | 335 |
| Nordic | 13 | 3466 | 267 | 128 | 200 | 350 | 220 |
| BeNeFrance | 33 | 6535 | 198 | 193 | 60 | 315 | 131 |
| UK/IRL | 9 | 1762 | 196 | 153 | 100 | 176 | 166 |
| South Europe | 62 | 7214 | 116 | 58 | 70 | 169 | 101 |
| East Europe | 12 | 1114 | 93 | 49 | 53 | 122 | 100 |
| **TOTAL** | **147** | **27223** | **185** | **268** | **77** | **194** | **138** |

BeNeFrance, Belgium, France, Luxemburg, the Netherlands; DACH, Germany (D), Austria (A), Switzerland (CH); IQR, Interquartile range; SD, standard deviation; UK/IRL, Republic of Ireland (IRL), United Kingdom (UK)
