# Supplementary material for: Contemporary European practice in transcatheter aortic valve implantation: results from the 2022 European TAVI Pathway Registry
Source: Front Cardiovasc Med. 2023 Aug 14;10:1227217. doi: 10.3389/fcvm.2023.1227217 (PMC10461475; doi:10.3389/fcvm.2023.1227217)
Supplement: Supplementary file 5 [file Table5.docx]

**Supplemental Table 5.** TAVI case per operator per region.

|  | Total Operators | Mean  Cases per Operator | ±SD | IQR1 | IQR3 | MEDIAN |
| --- | --- | --- | --- | --- | --- | --- |
| DACH | 84 | 97 | 49 | 66 | 124 | 87 |
| Nordic | 62 | 59 | 35 | 41 | 58 | 47 |
| BeNeFrance | 131 | 48 | 30 | 24 | 70 | 39 |
| UK/IRL | 29 | 62 | 27 | 44 | 70 | 58 |
| South Europe | 196 | 41 | 23 | 25 | 51 | 34 |
| East Europe | 38 | 32 | 22 | 20 | 36 | 29 |
| **Total** | **540** | **52** | **35** | **26** | **69** | **43** |

BeNeFrance, Belgium, France, Luxemburg, the Netherlands; DACH, Germany (D), Austria (A), Switzerland (CH); IQR, Interquartile range; SD, standard deviation; UK/IRL, Republic of Ireland (IRL), United Kingdom (UK)
