# Supplementary material for: Contemporary European practice in transcatheter aortic valve implantation: results from the 2022 European TAVI Pathway Registry
Source: Front Cardiovasc Med. 2023 Aug 14;10:1227217. doi: 10.3389/fcvm.2023.1227217 (PMC10461475; doi:10.3389/fcvm.2023.1227217)
Supplement: Supplementary file 6 [file Table6.docx]

**Supplemental Table 6.** Waiting times per centre size, per region and per organization type, in percentages.

|  | <2 weeks | <3months | 3-6 months | >6 months | Strongly depending private vs. public |
| --- | --- | --- | --- | --- | --- |
| **Centre size** | | | | |  |
| <50 | 0 | 73 | 20 | 7 | 0 |
| 50-99 | 5 | 78 | 8 | 5 | 3 |
| 100-199 | 8 | 75 | 14 | 4 | 0 |
| 200-499 | 17 | 60 | 17 | 6 | 0 |
| >499 | 22 | 67 | 11 | 0 | 0 |
| **Region** | | | | |  |
| DACH | 28 | 72 | 0 | 0 | 0 |
| Nordic | 25 | 46 | 38 | 8 | 0 |
| BeNeFrance | 12 | 82 | 6 | 0 | 0 |
| UK/IRL | 0 | 78 | 22 | 0 | 0 |
| South Europe | 6 | 73 | 13 | 6 | 2 |
| East Europe | 0 | 55 | 27 | 18 | 0 |
| **Structured Pathway** | | | | | |
| Yes | 9 | 71 | 14 | 5 | 1 |
| No | 12 | 71 | 12 | 6 | 0 |
| **Heart Valve outpatient Clinic** | | | | | |
| Yes | 9 | 72 | 14 | 4 | 1 |
| No | 10 | 70 | 12 | 7 | 0 |
| **Paramedical staff** | | | | | |
| Yes | 9 | 70 | 16 | 4 | 1 |
| No | 10 | 72 | 12 | 6 | 0 |

BeNeFrance, Belgium, France, Luxemburg, the Netherlands; DACH, Germany (D), Austria (A), Switzerland (CH); UK/IRL, Republic of Ireland (IRL), United Kingdom (UK)
