# Supplementary material for: Contemporary European practice in transcatheter aortic valve implantation: results from the 2022 European TAVI Pathway Registry
Source: Front Cardiovasc Med. 2023 Aug 14;10:1227217. doi: 10.3389/fcvm.2023.1227217 (PMC10461475; doi:10.3389/fcvm.2023.1227217)
Supplement: Supplementary file 7 [file Table7.docx]

**Supplemental Table 7.** Type of anaesthesia per centre size and region, in percentages.

|  | General Anaesthesia | Conscious sedation | Local anaesthesia |
| --- | --- | --- | --- |
| **Centre size** | | | |
| <50 | 7 | 87 | 7 |
| 50-99 | 14 | 57 | 30 |
| 100-199 | 6 | 67 | 27 |
| 200-499 | 3 | 46 | 51 |
| >499 | 0 | 44 | 56 |
| **Region** | | | |
| DACH | 0 | 67 | 33 |
| Nordic | 0 | 8 | 92 |
| BeNeFrance | 9 | 61 | 30 |
| UK/IRL | 0 | 44 | 56 |
| South Europe | 11 | 66 | 23 |
| East Europe | 0 | 83 | 17 |

BeNeFrance, Belgium, France, Luxemburg, the Netherlands; DACH, Germany (D), Austria (A), Switzerland (CH); UK/IRL, Republic of Ireland (IRL), United Kingdom (UK)
