# Supplementary material for: Contemporary European practice in transcatheter aortic valve implantation: results from the 2022 European TAVI Pathway Registry
Source: Front Cardiovasc Med. 2023 Aug 14;10:1227217. doi: 10.3389/fcvm.2023.1227217 (PMC10461475; doi:10.3389/fcvm.2023.1227217)
Supplement: Supplementary file 8 [file Table8.docx]

**Supplemental Table 8.** Alternative Access for TAVI. (%)

|  | **Trans femoral** | | **Trans Axillary** | | **Trans Thoracic** | | **Trans Carotid** | | **Trans Venous** | |
| --- | --- | --- | --- | --- | --- | --- | --- | --- | --- | --- |
|  | Balloon Angioplasty | Lithotripsy | Direct Percutaneous | Surgical Cutdown | Transapical | Direct Aortic | Direct Percutaneous | Surgical Cutdown | Transcaval | Transseptal |
| First Choice | 78 | 22 | 23 | 77 | 80 | 20 | 0 | 100 | 57 | 43 |
| Second Choice | 28 | 72 | 29 | 71 | 67 | 33 | 0 | 100 | 50 | 50 |
| Third Choice | 45 | 55 | 42 | 58 | 56 | 44 | 17 | 83 | 100 | 0 |
| **TOTAL** | **54** | **46** | **35** | **65** | **64** | **36** | **6** | **94** | **69** | **31** |
